# Amsler grid test for detection of advanced glaucoma in Ethiopia

**DOI:** 10.1371/journal.pone.0230017

**Published:** 2020-03-10

**Authors:** Girum W. Gessesse, Lemlem Tamrat, Karim F. Damji

**Affiliations:** 1 Department of Ophthalmology, St Paul’s Hospital Millennium Medical College, Addis Ababa, Ethiopia; 2 Department of Ophthalmology and Visual Sciences, University of Alberta, Edmonton, Canada; LV Prasad Eye Institute, INDIA

## Abstract

**Objective:**

This study was done to determine the validity of amsler grid test black on white (BOW), as well as white on black (WOB) for identifying central visual field (VF) defects in patients with advanced glaucoma.

**Design:**

Prospective study.

**Participants:**

We prospectively included 100 consecutive eyes of 88 adult patients with advanced glaucoma and 100 eyes of 100 normal individuals. We used a lottery method to choose the side of the eye for the control groups.

**Methods:**

All participants had reliable Humphrey 10–2 Swedish Interactive Threshold Algorithm (SITA) standard VF. Both the BOW and WOB amsler grid tests were done for each group. Sensitivity, specificity, and positive and negative predictive values of the amsler grid scotoma area were calculated with the 10–2 VF as the reference standard.

**Results:**

The mean ± standard deviation (SD) of age and the 10–2 VF mean deviation (MD) of advanced glaucoma eyes were 59.8 ± 11.8 (range 34–84) years and -19.94 ± 9.8(range -34.98–-0.52) respectively. Among 108 eyes with normal 10–2 VF test, 103 had a normal BOW amsler grid test and 5 had an abnormal BOW test. Among 92 eyes with an abnormal 10–2 VF test, 74 had an abnormal and 18 had normal BOW amsler grid test. Sensitivity, specificity, and positive and negative predictive values of the BOW amsler grid test were 80.4%, 95.4%, 93% and 85.1% respectively whereas that of the WOB amsler grid test were 71.7%, 95.4%, 93% and 72.8% respectively.

**Conclusion:**

The sensitivity and specificity of both BOW and WOB amsler grid tests were high in detecting VF defects in advanced glaucoma.

## Introduction

Glaucoma is the leading cause of irreversible blindness worldwide[1], and the second cause of blindness (after cataract) from various conditions in Sub-Saharan Africa (SSA)[2]. Clinic based studies in Africa have shown that 60%–76% of patients with glaucoma present in advanced stage of the disease or unilateral blindness[3].

Early detection is important to reduce the risk of advanced glaucomatous disease and loss of quality of life and productivity. Early in the disease process, patients with glaucoma are asymptomatic and central vision and the central visual field (VF) is typically intact. In advanced stage the remaining central island of vision is in imminent danger of loss, particularly in SSA settings where detection and treatment are far from optimal. Detecting glaucoma at this stage is of utmost importance since aggressive treatment can prevent blindness[4]. Central vision is more important for patients than peripheral VF loss and visual acuity is strongly related to VF sensitivity in the central area of the 10–2 VF[5]. Tasks involving central and near vision (i.e. reading) have been given the greatest importance, followed by mobility outside the home (i.e. driving and walking outside) and the relative emphasis of these priorities changed with increasing VF loss, with concerns for central vision increasing, whereas those for outdoor mobility decreased[6]. If patients can be diagnosed in advanced stage, while they still have the central vision intact and treated, it has multiple benefits in terms of personal, social and economic factors and is likely to maintain a reasonable quality of life.

The sensitivity and specificity of automated white on white perimetry has been reported to be 70% and 67%, respectively[7]. The performance of frequency doubling technology (FDT) perimetry has been evaluated extensively in population based glaucoma prevalence surveys where the sensitivity and specificity values for detecting definite glaucoma was reported to be 55.6% and 92.7%, respectively[8]. Various studies have also shown that the sensitivity increases up to 96.6% in those eyes with mean deviation (MD) of less than -8 dB[8,9]. However, the equipment needed for such perimetry is expensive, requires expertise for interpretation and is not easily available in the developing countries like Ethiopia. This limits their utilization in community setting.

The amsler grid was designed specifically to analyze VF defects in the central 10 degrees surrounding fixation. It is a high contrast supra-threshold test. It is used to detect and monitor central field defects for macular diseases[10,11]. It is a valuable test due to the low cost, ease of administration, short testing and process time, and ease of understanding and performance for the patient and no need of maintenance.

There is scarcity of data on the use of amsler grid test for detection of glaucoma. A recent Hospital based study in USA showed high sensitivity and specificity of the black on white (BOW) test to detect central vision loss from moderate to severe glaucoma[12].

This study was done primarily to determine the validity of amsler grid test for identifying central field defect in patients with advanced glaucoma in Ethiopia.

## Materials and methods

### Study population

This was a cross sectional study undertaken between March 2016 to March 2018 at St.Paul’s Hospital Millennium Medical College(SPHMMC), Addis Ababa, Ethiopia. We prospectively included consecutive eyes of patients with advanced glaucoma and eyes of normal adults with no glaucoma visiting the department during the study period. Both eyes were included for 12 patients with advanced glaucoma disease. Inclusion criteria for glaucoma cases included documented evidence of advanced stage (defined below) who had a reliable 10–2 SITA standard VF test (Humphrey Field Analyzer II; Carl Zeiss Meditec, Inc., Dublin, CA stimulus size Goldman III) within the preceding 3 months. Reliability was defined as, fixation loss or false positive <20%, and false negative <30%.

We excluded patients if they had systemic or ocular diseases other than glaucoma known to affect the VF, history of use of hydroxychloroquine, tamoxifen, digoxin, central/paracentral corneal pathology and significant cataract, macular diseases, posterior segment intraocular surgery, and best corrected visual acuity (BCVA) less than 20/200.

The normal group included individuals >18 years of age with BCVA better than 6/12, normal IOP, and no evidence of glaucomatous optic neuropathy, and a normal VF test on 10–2 HVF. We employed a lottery method to choose the side of the eye for the control groups.

A structured data collection format was designed for the purpose of the study and all relevant clinical information was recorded. Clinical evaluation included BCVA, slit-lamp biomicroscopy and stereoscopic optic nerve head examination using + 90D Volk lens by GG and LT who are fellowship trained and a glaucoma fellow, respectively.

The amsler grid tests were done by senior resident and senior optometrists who are not involved in the care of the patient, and who were masked to the patient’s clinical exam findings and VF. After correcting for near refractive error, each participant was asked to view each amsler grid (10&10 cm) at a distance of 30 cm, ensuring that each box on the amsler grid corresponds to 1 degree of VF. A similar high quality paper copy of the amsler grid was used for each patient. An eye patch was used to occlude the eye not being tested. Patients were instructed to fixate on the central point of the grid at all times. The patient was then asked to draw out the borders of any perceived scotomas (missing or blurry areas) on the grid. Patients perform this amsler test prior to getting a thorough eye examination because applanation and dilation could affect their performance. We used the same examination room with the same lighting conditions for all patients.

The study respected the tenets of ethical declaration of Helsniki. Ethical clearance was obtained from the Institutional Review Board(IRB) of saint Paul’s Hospital millennium medical college. Verbal Informed consent was obtained from individual patients and individual information of patients was kept confidential.

### Definitions of terms

Advanced glaucoma was defined as advanced glaucomatous disc features (e.g., C/D ≥ 0.9) and (or) VF defect within 10° of fixation [13]. Amsler grid test was considered abnormal if a patient had any perceived scotoma with any amount of missing or blurry grid lines inside the chart. The affected area on the amsler grid test chart was calculated by taking each square as equivalent to 1 degree.

An abnormal 10–2 VF was defined as the presence of at least 3 contiguous test points within the same hemifield on the pattern deviation plot at P < 0.01, with at least 1 point at P <0.005. Among eyes with abnormal 10–2 VF test, the MD, scotoma extent (SE) and the scotoma mean depth (SMD) were calculated using a similar study[12]. The number of abnormal test points with P <0.01 on the total deviation map was recorded and defined as SE (10–2). The average total deviation sensitivity (dB) of points with P<0.01 on the total deviation map was defined as SMD.

We classified the hemifield locations of the amsler grid scotomas and the 10–2 VF scotomas into 3 categories: (1) predominantly in the superior hemifield, (2) predominantly in the inferior hemifield, and (3) equivocal. The hemifield location of the amsler grid scotoma was determined by comparing the scotoma area between superior and inferior hemifields. The hemifield location of a 10–2 VF scotoma was determined by comparing the average total deviation sensitivity between superior and inferior hemifields. If the difference in the amsler grid scotoma area or the average total deviation sensitivity of 10–2 VF between superior and inferior hemifields was less than 5% of the worse value or less than 1 dB between the 2 hemifields, the scotoma was considered as equivocal.

### Statistical methods

Sensitivity, specificity, positive predictive value and negative predictive value of amsler grid tests were calculated by taking 10–2 HVF test as a standard. To determine the relationship between amsler grid scotoma and 10–2 VF scotoma, linear and quadratic regression analysis were done between amsler grid scotoma area and 10–2 VF parameters (MD, SE, and SMD) among eyes with abnormal 10–2 VF defect. Commercially available statistical software (SPSS version 17.0 for Windows; SPSS Inc., Chicago, IL) was used for all analyses. A P value <0.05 was considered significant.

## Result

The mean ± standard deviation (SD) of age and the 10–2 VF MD of advanced glaucoma eyes were 59.8±11.8 (range 34–84) years and -19.94±9.8 (range -34.98–-0.52) dB respectively and 63% were males. The mean ± standard deviation age and the 10–2 VF MD of normal individuals were 43.4 ± 14.0 (range 18–76) years,–0.8 ± 0.9 (-1.2–1.5) dB and 52% were men. Among advanced glaucoma(AG) patients, 53(53%) were POAG, 37 (37%) pseudoexfoliative glaucoma, 8(8%) were chronic angle closure glaucoma (CACG).

Among 108 eyes with normal 10–2 VF test, 103 had a normal BOW amsler grid test and 5 had an abnormal BOW amsler grid test. Among 92 eyes with an abnormal 10–2 VF test, 74 had an abnormal and 18 had normal BOW amsler grid test. (Table 1).

**Table 1:** 10–2 Humphrey SITA standard visual field test and black on white amsler grid test results among 200 eyes.

		10–2 VF (No. of eyes)		Total
		Normal	Abnormal	
BOW amsler test(No. of eyes)	Normal	103	18	121
	Abnormal	5	74	79
Total		108	92	200

VF = visual field; BOW = black on white

On the other hand, from the 108 eyes with normal 10–2 VF test, 103 had a normal and 5 had an abnormal WOB amsler grid test. Among 92 eyes with an abnormal 10–2 VF test, 66 had an abnormal and 26 had normal WOB amsler grid test. (Table 2).

**Table 2:** 10–2 Humphrey SITA standard visual field test and white on black amsler grid test results among 200 eyes.

		10–2 VF (No. of eyes)		Total
		Normal	Abnormal	
WOB amsler test (No. of eyes)	Normal	103	26	129
	Abnormal	5	66	71
Total		108	92	200

VF- visual field; WOB = white on black

Among 92 patients who had abnormal 10–2 HVF test, there was no statistically significant difference in the mean visual acuity (VA) between those with normal BOW amsler test results and abnormal results (mean VA = 0.6 and 0.5 respectively, P = 0.17).

Both BOW and WOB amsler grid test’s scotoma area was significantly associated with 10–2 VF MD, and SMD (P-value <0.001, chi-square test)). Sensitivity, specificity, and positive and negative predictive values of the BOW amsler grid test were 80.4%, 95.4%, 93% and 85.1% respectively whereas that of the WOB amsler grid test were 71.7%, 95.4%, 93% and 72.8% respectively.

For the 74 eyes with abnormal BOW amsler grid test and 10–2 VF, there was no correlation of the hemifield location of the amsler grid scotoma and that of the 10–2 VF scotoma (cramer’s correlation coefficient V = 0.183, P = 0.290). The finding was not different after the equivocal ones were excluded (cramer’s correlation V = 0.115, P = 0.32)(Table 3). The finding was also the same for WOB amsler grid test and 10–2 VF test (Cramer’s correlation coefficient V = 0.169, P = 0.437) (Table 4).

**Table 3:** Hemifield location of scotomas of 10–2 VF test vs amsler BOW test in advanced glaucoma eyes when both tests were abnormal.

		10–2 VF (No. of Eyes)			Total
		Predominantly superior	Predominantly inferior defect	Equivocal	
Amsler Grid (BOW)	Predominantly superior	17	5	3	25
	Predominantly inferior	12	8	2	20
	Equivocal	14	9	7	29
Total		43	22	12	74

VF = visual field. BOW = black on white

The finding was also the same for WOB amsler grid test and 10–2 VF test (Cramer’s correlation coefficient V = 0.169, P = 0.437) (Table 4).

**Table 4:** Hemifield location of scotomas of 10–2 VF test vs amsler WOB test in advanced glaucoma eyes when both tests were abnormal.

		10–2 VF (No. of Eyes)			Total
		Predominantly superior	Predominantly inferior defect	Equivocal	
Amsler Grid (WOB)	Predominantly superior	7	6	3	16
	Predominantly inferior	14	8	5	27
	Equivocal	22	7	4	33
Total		43	21	12	76

VF = visual field. WOB = White on black

**Fig 1:**
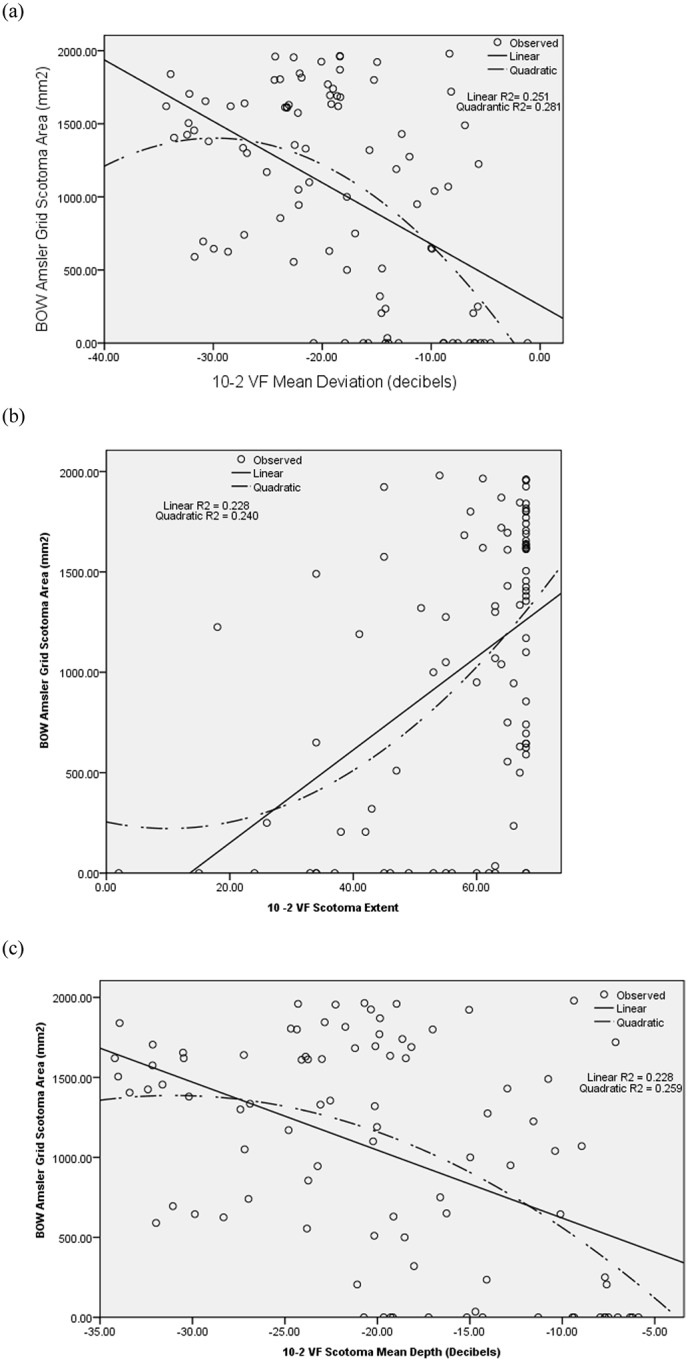
The relationship among the amsler grid scotoma area (BOW) and 10–2 VF mean deviation (MD). (A), scotoma extent (SE) (B), and scotoma mean depth(C) in 92 glaucomatous eyes with abnormal 10–2 VF test.

The pearson’s correlation coefficient shows significant weak correlation among the BOW amsler grid scotoma area with the 10–2 VF MD (linear R^2^ = 0.251, quadratic R^2^ = 0.281, P<0.001), 10–2 VF scotoma extent(linear R^2^ = 0.228 = quadratic R^2^ = 0.240, P < 0.001), and 10–2 VF SMD (linear R = 0.228, quadratic R = 0.259, P<0.001) in 92 glaucomatous eyes with abnormal 10–2 VF test, r = -0.501, p-value = <0.001, r = 0.478, p-value = <0.001 and r = -0.478, p-value = <0.001 respectively. Similarly there was weak correlation among the WOB amsler grid scotoma area and the 10–2 visual field MD (A), SE (B) and SMD (C) in 92 glaucomatous eyes with abnormal 10–2 VF test result, (pearson’s correlation coefficient r = -0.507, P <0.001; r = 0.482, P<0.001 and r = -0.488, P<0.001 respectively). The liner and quadratic regression among the BOW amsler grid scotoma area and the10-2 VF MD showed significant relationship (P<0.001 and 0.03 respectively). The liner regression among the BOW amsler grid scotoma area and the 10–2 VF SE showed significant relationship (P<0.001) but quadratic regression was not significant (P = 0.20). Likewise the liner regression among the BOW amsler grid scotoma area and the 10–2 VF SMD showed significant relationship (P<0.001) but quadratic regression was borderline (P = 0.058)(Fig 1).

**Fig 2:**
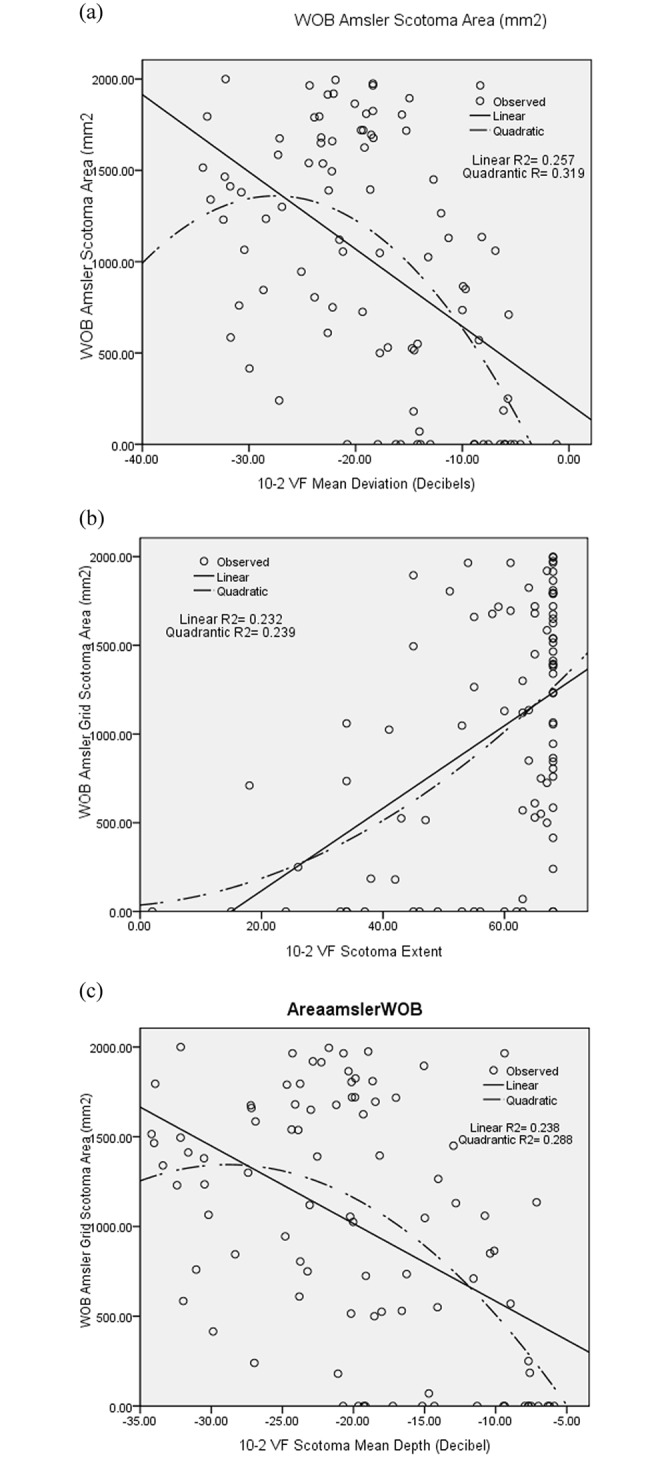
The relationship among the amsler grid scotoma area (WOB) and 10–2 VF mean deviation (MD). (A), scotoma extent (SE) (B), and scotoma mean depth(C) in 92 glaucomatous eyes with abnormal 10–2 VF test.

The WOB amsler grid scotoma area had the strongest relationship with 10–2 MD (quadratic R^2^ = 0.319), followed by 10–2 SE (quadratic R^2^ = 0.239) and 10–2 SMD (quadratic R^2^ = 0.288) (all P < 0.001 except for 10–2 SE p-value = 0.384)(Fig 2).

## Discussion

The amsler grid has already been demonstrated as an effective tool in accurately evaluating, characterizing and monitoring VF defects in patients with age-related macular degeneration (AMD)[11,14]. However, it is not commonly used as a clinical tool for detection of VF defects in glaucoma, and in particular for advanced glaucoma.

In this study, 10–2 HVF test was taken as a clinical standard reference to detect advanced glaucoma and the sensitivity and specificity of amsler grid test was found to be 80.4%, 95.4% for BOW and 71.7%, 95.4% for WOB amsler grid tests, respectively. A study in the USA among early to advanced stages of glaucoma found the sensitivity, and specificity of the BOW amsler grid test to be 68% and 92%, respectively, but the sensitivity increased from 40% in eyes with 10–2 MD better than—6 dB to 92% in eyes with 10–2 MD worse than -12 dB[12].

The specificity and positive predictive value (PPV) of the BOW and WOB amsler grid test was 95.4%, 95.4% and 95.4%, 95.4% respectively; this specificity is higher than the USA study that was 92%[16]. Both BOW and WOB amsler grid test’s scotoma area was significantly associated with 10–2 VF MD, SE, and SMD (P <0.0001). This suggests that amsler grid test can likely be used in detecting advanced cases and possibly monitoring glaucoma progression in those individuals with central VF defect, although additional studies are needed to examine this possibility.

There was no correlation of the hemifield location of these two tests. This result was different from a previous study[12]. The reason for this could be the definition used for the two tests. In case of amsler test we use scotoma area average total deviation sensitivity was used for the 10–2 test and these two may not exactly correspond.

To correlate amsler grid test results with those of 10–2 VF test in the previous study, a circle was drawn reflecting central 10 degrees of VF on each grid, while we used the original square amsler chart; this may have created a disparity in results between the studies.

**Fig 3:**
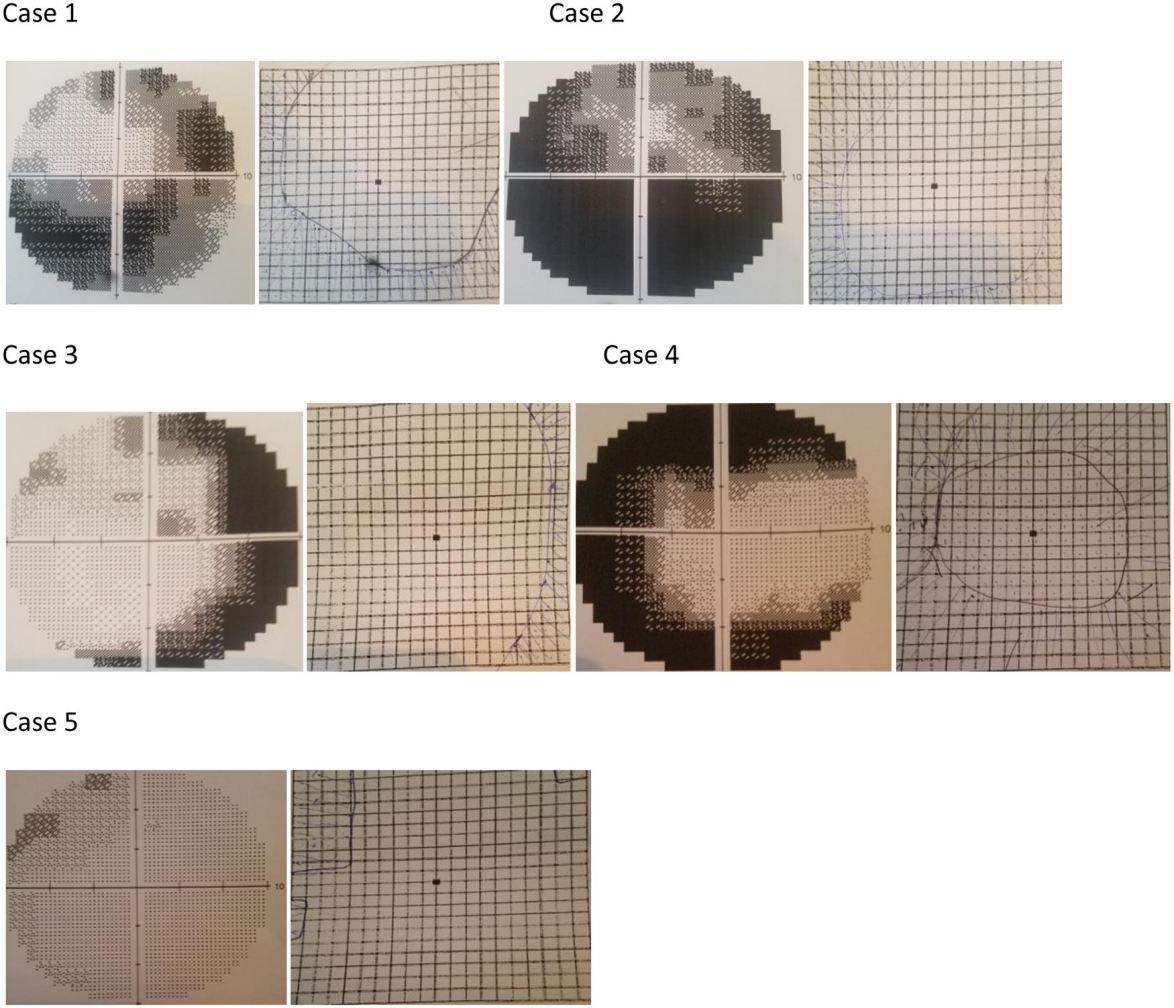
Representative amsler grid test results with corresponding 10–2 visual field [VFs].

A few representative cases showing correlation between amsler grid and 10–2 VF are shown in Fig 3.

In population screening it is also reported that high sensitivity of a test is of greatest significance in cases involving advanced stages of the disease including glaucoma[15]. According to our study, the amsler grid test would be acceptable and appropriate in this regard as it has high sensitivity and specificity. Furthermore, the test is inexpensive, safe and simple to perform, and is likely to be very acceptable to a high risk population that is screened. It can also be used in large community-based screening programmes in the primary care setting for widespread screening of advanced glaucoma patients. The simplicity of the test may have an added benefit of enhancing patient understanding of their disease[12]. It can also provide patients an opportunity to understand the impact of their glaucomatous visual field loss, and this may improve their adherence to medications[16].

The sensitivity of amsler grid test in detecting scotomas of various eye diseases has been reported to be 19%, but only 7.3% of study patients had glaucoma[17]. In another study the sensitivity of amsler grid test in detecting AMD ranged from 9% to 34%, the latter being the advanced AMD cases[18,19]; this is inferior to our result for advanced glaucoma, suggesting amsler grid test can better detect advanced glaucoma than advanced AMD.

Recent advance in technology have brought about computerization of threshold amsler grid testing to detect visual field defect in early or suspect glaucoma cases[20]. Although this test shows promise, it is not widely available and it requires patients to be present in an office equipped with the test device or at least may require a portable computer, and therefore does not offer the accessibility and simplicity that the paper test offers.

Central visual field testing in glaucoma is of paramount importance because the macula includes approximately 30% of all retinal ganglion cells (RGCs)[21] and damage to the macula can substantially affect health-related quality of life (QOL). Glaucomatous damage to the macula maybe missed in routine examinations if only standard 24–2 VF is performed. The use of 10–2 VF test has shown high prevalence of central defects among patients with early glaucoma; with 61.5% of eyes classified as normal on 24–2 tests were classified as abnormal on 10–2 visual fields[22–24].

### Limitations

The small number of patients included is the main limitation of the study. Inclusion of patients with different stages of glaucoma may also be worthwhile to show the performance of the test during various phases of the natural history. Additionally not taking age matched controls might have affected the result too. The fact that we did not draw a circle on the amsler test to correspond with the 10–2 VF area may also have affected some of our results in comparing the amsler to the 10–2 field.

## Conclusion

Both BOW and WOB amsler Grid tests are sensitive and specific in detecting VF defects in advanced glaucoma and can thus be used for screening. The BOW test shows slight better result than the WOB Since most of our patients have access to primary eye care centers where there is no formal VF testing available, both types of testing can assist in detecting advanced glaucoma cases which can be referred for further investigation and treatment. Further large scale studies needs to be done so that this test can be used in community based screening programmes.

## Supporting information

S1 Data (SAV)
